# Presolvated Electron Reactions with Methyl Acetoacetate: Electron Localization, Proton-Deuteron Exchange, and H-Atom Abstraction

**DOI:** 10.3390/molecules190913486

**Published:** 2014-09-01

**Authors:** Alex Petrovici, Amitava Adhikary, Anil Kumar, Michael D. Sevilla

**Affiliations:** Department of Chemistry, Oakland University, Rochester, MI 48309, USA; E-Mails: aopetrov@oakland.edu (A.P.); adhikary@oakland.edu (A.A.); kumar@oakland.edu (A.K.)

**Keywords:** prehydrated electron, anion radical, proton-deuteron exchange, H-atom abstraction

## Abstract

Radiation-produced electrons initiate various reaction processes that are important to radiation damage to biomolecules. In this work, the site of attachment of the prehydrated electrons with methyl acetoacetate (MAA, CH_3_-CO-CH_2_-COOCH_3_) at 77 K and subsequent reactions of the anion radical (CH_3_-CO•^−^-CH_2_-COOCH_3_) in the 77 to ca. 170 K temperature range have been investigated in homogeneous H_2_O and D_2_O aqueous glasses by electron spin resonance (ESR) spectroscopy. At 77 K, the prehydrated electron attaches to MAA forming the anion radical in which the electron is delocalized over the two carbonyl groups. This species readily protonates to produce the protonated electron adduct radical CH_3_-C(•)OH-CH_2_-COOCH_3._ The ESR spectrum of CH_3_-C(•)OH-CH_2_-COOCH_3_ in H_2_O shows line components due to proton hyperfine couplings of the methyl and methylene groups. Whereas, the ESR spectrum of CH_3_-C(•)OH-CH_2_-COOCH_3_ in D_2_O glass shows only the line components due to proton hyperfine couplings of CH_3_ group. This is expected since the methylene protons in MAA are readily exchangeable in D_2_O. On stepwise annealing to higher temperatures (*ca.* 150 to 170 K), CH_3_-C(•)OH-CH_2_-COOCH_3 _undergoes bimolecular H-atom abstraction from MAA to form the more stable radical, CH_3_-CO-CH•-COOCH_3_. Theoretical calculations using density functional theory (DFT) support the radical assignments.

## 1. Introduction

Ionizing radiation transfers energy to matter by the production of holes, ejected electrons, and excited states [1]. Of these, the holes have been considered to be the most damaging [1]. However, in the last few years, electrons, especially low energy electrons (LEEs), have been recognized as a major contributor to radiation damage [2,3]. LEEs are simply electrons having energies in the range of 0–20 eV which can cause damage by inducing bond cleavage via dissociative electron attachment (DEA) [2,3]. In aqueous solution, the LEEs that do not react by DEA, undergo thermal deactivation and lead to the formation of solvated electrons (e_aq_^−^) [2,3]. e_aq_^−^ add to the electron affinic sites in molecules and induce reactions that follow adiabatic pathways [3]. 

Electrons formed in irradiated LiCl aqueous glasses are predominantly trapped in shallow wells [4]. The most abundant trapped electron species is *ca.* 0.5 eV below the continuum, although some traps can be as deep as 2.6 eV [4]. Since electrons react with the solutes prior to complete solvation, the reactions of electrons in these irradiated glasses are primarily due to these partially solvated electrons which are known as presolvated (prehydrated) electrons (e_pre_^−^) [5,6,7].

**Scheme 1:**
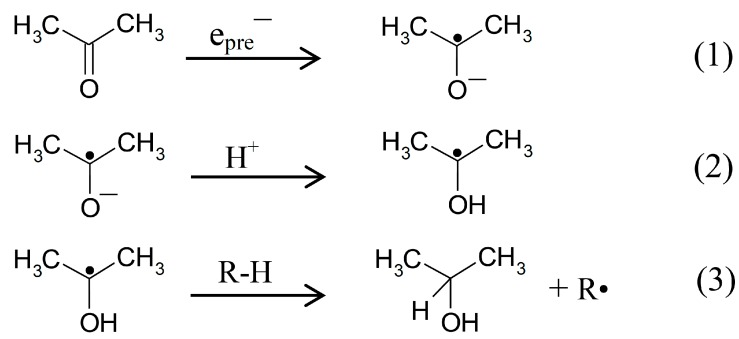
Reactions intiated by presolvated electron attachment to acetone.

For many molecular systems with functional groups that can capture electrons such as ketones, esters, carboxylic acids, and peptides, it is well known that the anion radicals formed on e_pre_^−^ addition undergo subsequent reactions [8,9,10,11]. For peptides, the most common reaction of the anion radical is either deamination or deamidation. For ketones and esters, the anion radicals formed on electron attachment lead to reactive intermediates that may undergo bimolecular H-atom abstraction reactions. This is shown below for acetone anion radical, p*K*_a_ = *ca.* 12 [12] (Scheme 1, reaction (1)), which after protonation (Scheme 1, reaction (2)) produces a neutral C-centered radical; this radical can undergo bimolecular H-atom abstraction from a weak C-H bond to form isopropyl alcohol (Scheme 1, reaction (3)) [8].

**Scheme 2:**
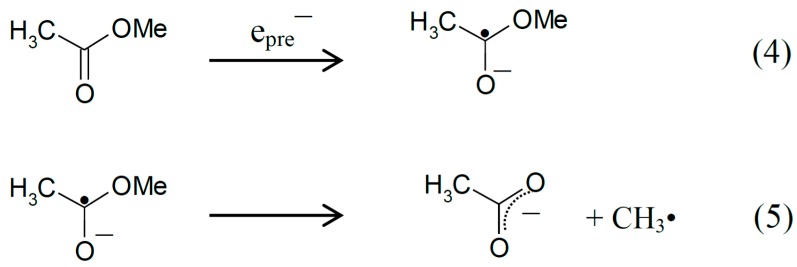
Reactions initiated by presolvated electron attachment to methyl acetate.

Or, for esters, O-C bond cleavage reactions (Scheme 2, reactions (4, 5)) by DEA in the anion radical formed after the addition of the prehydrated electron (e_pre_^−^) to the carbonyl group, are observed frequently [9], such as:

In the current study, we have investigated prehydrated electron attachment to methyl acetoacetate (MAA) in homogeneous aqueous (H_2_O or D_2_O) glasses at 77 K and followed the subsequent reactions of the MAA anion radical in the 77 to *ca.* 170 K temperature range employing electron spin resonance (ESR) spectroscopy and density functional theory (DFT). We note that irradiation of 7.5 M LiCl aqueous glasses at 77 K produces only two intermediates: prehydrated electrons and Cl_2_•^−^ [10,13,14]. In our system Cl_2_•^−^ is unable to one-electron oxidize simple aliphatic ketones or esters and thus does not interfere with the study of the reactions of prehydrated electron [10]. The combination of experimental and theoretical results clearly shows that in MAA the initial localization of the electron is chiefly at the carbonyl site and that protonation of the carbonyl group further localizes the electron to this site. The protonated MAA anion radical is found to undergo bimolecular H-atom abstraction from the parent compound (*i*.*e*., MAA). 

## 2. Results and Discussion

### 2.1. ESR Studies in H_2_O

**Figure 1:**
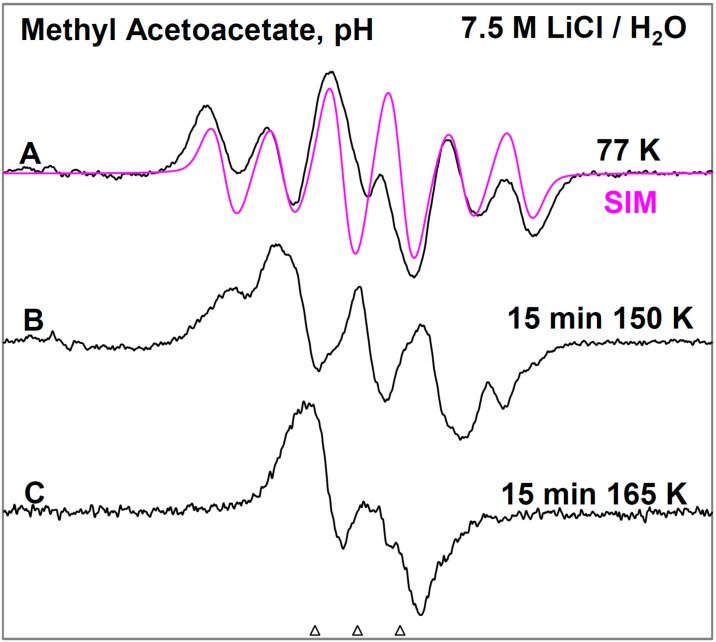
ESR spectra of the prehydrated electron addition to MAA at 77 K and subsequent reactions of the MAA anion radical on annealing to 165 K in a 7.5 M LiCl H_2_O glass. (**A**) The black spectrum of the protonated MAA anion radical (radical II). The pink spectrum is the simulated spectrum of radical II. For simulation parameters, see text; (**B**) Radical II, after conformational rearrangement; (**C**) Radical III, CH_3_-CO-C•H-CO-OCH_3_, formed by H-atom abstraction from the parent MAA molecule by radical II at 165 K. The three reference markers (open triangles) in this figure and in the subsequent figures show the position of Fremy’s salt resonance with the central marker at g = 2.0056. Each of these markers is separated from each other by 13.09 G.

In Figure 1, the ESR spectra found after γ-irradiation (600 Gy) at 77 K of a sample of MAA in 7.5 M LiCl in H_2_O are shown. The black spectrum in Figure 1A resulting from electron attachment to MAA clearly shows a six line multiplet which is assigned to three *ca.* 18 G proton hyperfine couplings from a methyl group and a single proton coupling of *ca.* 37 G from one of the methylene group protons. The experimental spectrum (black) has been simulated using four lines of equal intensity separated by 18 G to account for the methyl proton hyperfine couplings, a single proton hyperfine coupling of 37 G, g-value = 2.0029, and a mixed ((Lorentzian/Gaussian) = 0.2) line-width = 8 G. The simulated spectrum (pink) has been superimposed on the experimental spectrum for comparison. This spectrum is assigned to radical II formed after protonation of the anion radical, reactions 6 and 7. The line intensities of the methyl protons are close to 1:1:1:1 rather than the expected 1:3:3:1 line intensities owing to the well-known tunneling rotation of the methyl groups at 77 K [15]. Such restricted rotation of methyl group is expected at low temperatures and has been observed after electron addition to CH_3_CO- groups such as in acetic acid, acetamide and N-acetylmethionine at 77 K [11,15,16]. These anion radicals exhibit the normal and expected 1:3:3:1 line intensity ratios of the methyl protons at higher temperatures (provided the anion radicals are stable at that elevated temperature [15]. Hyperfine couplings from both the methyl group and the methylene in spectrum 1A show that the electron addition is to the carbonyl group at 77 K. No evidence is found for electron addition to the acetate linkage of MAA even though esters are known to readily form anion radicals at low temperatures [9]. The trapping of the electron at the carbonyl group in MAA at 77 K might suggest that the carbonyl group in MAA has a higher electron affinity than its acetate moiety. However, DFT calculations presented here (Section 2.3) show that it is the protonation of the carbonyl oxygen in MAA that helps to localize the electron to the carbonyl group.

From the p*K*_a_ of the acetone anion radical, p*K*_a_ = *ca.* 12 [12], it is expected that the anion of carbonyl group in MAA anion radical has a p*K*_a_ less than 12 owing to delocalization of spin and charge (see Section 2.3). Thus, the carbonyl group of the MAA anion radical is expected to be protonated on warming; however, DFT calculations suggest that protonation has occurred even at 77 K and hence, we have assigned spectrum 1A to radical II. 

**Scheme 3:**
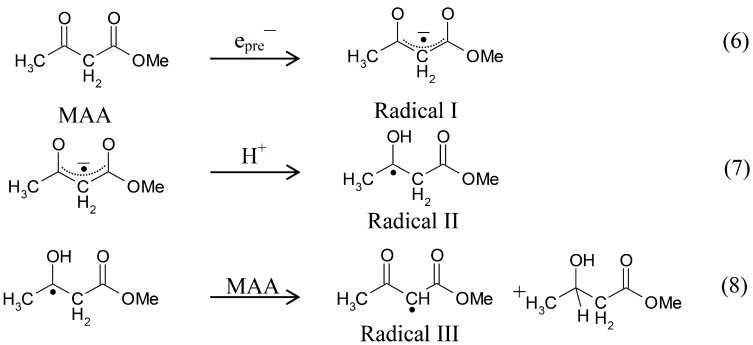
Reactions initiated by presolvated electron attachment to MAA.

On annealing from 77 K (spectrum 1A) to 150 K (spectrum 1B), we find that the spectrum changes substantially. Note that all spectra are recorded at 77 K after annealing at each temperature. The broad lines in Figure 1B make the spectral analyses difficult. However, it appears that in spectrum 1B, the methylene proton coupling drops from *ca.* 37 G to *ca.* 25 G (Table 1). Upon increasing the temperature from 77 to 150 K, the methylene protons likely take on a range of values that somewhat broaden the spectrum in Figure 1B. However, the methyl protons hyperfine couplings remain at 18 G (see spectrum 2B). These results are suggestive of a further change in the conformation of radical II on annealing from 77 K to 150 K. On this basis, spectrum 1B is assigned to the conformationally rearranged radical II (Scheme 3, reaction (7)).

**Table 1:** DFT Calculated Proton hyperfine couplings (B3LYP/6-31G*).

Radical	Phase	Proton Hyperfine Couplings ^1,2^ (Gauss)		
		CH_3_	CH_2_	O-H [xx, yy, zz] ^1^
I (opt)	vacuum	11.6	18.7, 6.6	-
	PCM	13.1	25.4, 4.9	-
II (opt)	vacuum	18.5	35.6, 0.8	[−6.5, −5.5, 5.4]
	PCM	18.7	35.9, 0.9	[−6.7, −5.7, 5.0]
II (Exp)	Glass ^3^	18	37, <5	unresolved
III (opt)	vacuum	C-H [xx, yy, zz]		-
		[−29.4, −20.7, −8.1]		-
	PCM	[−29.6, −20.9, −8.0]		-
III (Exp)	Glass ^3^	*ca.* −22		-

^1^ Hyperfine couplings are isotropic values except for those in brackets which are the sum of isotropic and anisotropic values; opt = geometry optimized; ^2^ Proton couplings for the methyl and methylene protons are β-proton couplings and have only small anisotropic components. Thus only the isotropic values are given;^ 3^ Glass = 7.5 M LiCl/H_2_O.

On annealing to 165 K (Figure 1C), we find that a *ca.* 22 G doublet (Table 1) builds in with the loss of the signal of the protonated electron adduct radical (II). This species is assigned to the radical (III) (CH_3_-CO-CH•-COOCH_3_) formed by the biomolecular H-atom abstraction of a methylene proton of the parent MAA molecule by the radical II (Scheme 3, reaction (8)). 

### 2.2. ESR Studies in D_2_O

Our results in D_2_O glasses, for otherwise identical (*i*.*e*., matched) samples clarify and add strong support for the above analyses (Scheme 3, reactions (6) to (8)). Owing to the p*K*_a_ of -CH_2_- group of MAA as 11 [17] and pD of the 7.5 M LiCl/D_2_O solution being *ca.* 5 [18], the CH_2_ group in MAA has undergone complete deuterium exchange in D_2_O solutions of 7.5 M LiCl *i*.*e*., MAA is converted to CH_3_-CO-CD_2_-COOCH_3_ in 7.5 M LiCl/D_2_O.

**Figure 2:**
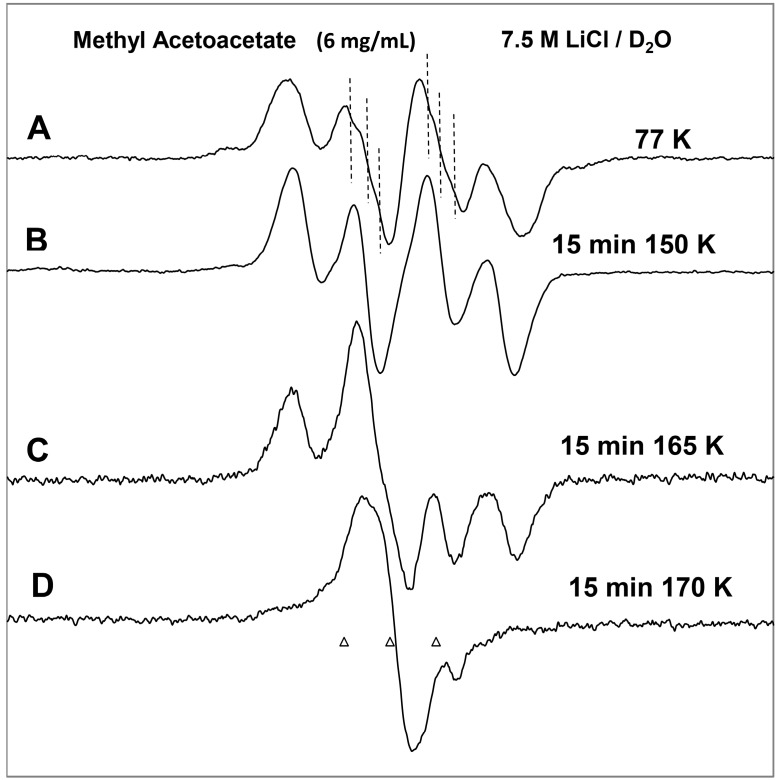
ESR spectra of prehydrated electron addition to MAA and subsequent reactions of the MAA anion radical on annealing to 170 K in a 7.5 M LiCl/D_2_O glass. (**A**) The deuterated electron adduct, radical II. Note the central components show distinct deuterium couplings of the exchanged methylene deuteron (marked with hash marks); (**B**) Deuterated radical II after conformational rearrangement in which the deuterium couplings are lost; (**C**) Gradual formation of deuterated radical III as radical II is lost; (**D**) Deuterated radical III, CH_3_-CO-C•D-COOCH_3,_ formed by hydrogen (deuteron) abstraction from the parent molecule by II at 170 K.

In Figure 2, the results in D_2_O glasses are shown. At 77 K, only four major lines expected from the methyl group hyperfine couplings (*ca.* 18 G) are observed in spectrum 2A. Owing to the smaller magnetic moment of deuterons than protons, deuterons show hyperfine couplings that are only 15% (1/6.514) that of protons in the same environment [18,19,20]. Hence, the one methylene proton coupling of *ca.* 37 G, is reduced to 5.7 G deuterium coupling. Also any unresolved couplings from the other remaining methylene proton and the hyperfine coupling due to –OH in radical II (Scheme 3, reaction (7)) are lost as expected since they are now deuterons in CH_3_-C•OD-CD_2_-COOCH_3_. Analyses of the central two lines in Figure 2A (77 K) do show a poorly resolved triplet (marked with lines) of 5.7 G as expected. Therefore, spectrum 2A is assigned to CH_3_-C•OD-CD_2_-COOCH_3_.

On annealing to 150 K, this poorly resolved triplet is lost in spectrum 2B which is expected since the rearrangement found in H_2_O at this temperature suggests only a 3.8 G deuteron coupling will be observed from the methylene, *i*.*e*., 25/6.514 G and this is too small to observe. However, the methyl proton hyperfine coupling remains at *ca.* 18 G in D_2_O as it was in H_2_O glasses.

On annealing to higher temperatures, 165 K (Figure 2C) and then to 170 K (Figure 2D), a singlet gradually builds in with the loss of the signal of four line spectrum of the deuterated radical II. This species is assigned to the deuterated radical III (reaction 8), *i*.*e*., CH_3_-CO-CD•-COOCH_3_, formed by deuterium atom abstraction from the deuterated methylene of the parent MAA (Scheme 3, reaction (8)). Comparison of Figure 1C (H_2_O) with Figure 2C (D_2_O) clearly shows that reaction (8) takes place more slowly in D_2_O over that found in H_2_O. This kinetic isotope effect is expected because a C-D bond is *ca.* 1 kcal/mole stronger than a C-H bond simply from vibrational zero point energy considerations [21].

In summary, the overall experimental results shown in Figure 1 and Figure 2 strongly support the following mechanism: prehydrated electron addition to the carbonyl group in MAA (Scheme 3, reaction (6)) which is followed by protonation of the electron adduct of MAA at 77 K (reaction 7) and the subsequent H-atom abstraction from the parent MAA by the protonated electron adduct (Scheme 3, reaction (8)).

The effect of pH has also been investigated in identically prepared samples in 7.5 M LiCl/H_2_O glasses but at pH *ca.* 8.5. The initial spectrum found at 77 K after the prehydrated electron addition shows the identical spectrum of the protonated electron adduct (II) found at pH 5. On annealing to 165 K, similar to the spectrum 1C, the *ca.* 22 G doublet is formed. Thus, the mechanism shown in reactions (6) to (8) is not affected by increasing the pH from *ca.* 5 to *ca.* 8.5.

### 2.3. DFT Calculations

DFT calculations for each of radical intermediates (I, II, and III) have been performed using the B3LYP functional and a 6-31G* basis set. The DFT/B3LYP/6-31G* method is known to provide hyperfine couplings that are in excellent agreement with the experimentally obtained ones [1,13,14,18,19,22]. In our calculations, the solvent has been treated by use of the polarized continuum model (PCM) as implemented in Gaussian 09 [23]. The geometry of each of the radicals has been optimized employing the DFT/B3LYP/6-31G* method and the hyperfine coupling constant values for each optimized radical intermediate were calculated. These theoretically calculated hyperfine coupling constants along with the corresponding experimentally obtained ones are shown in Table 1.

In radical I, the carbonyl group is not protonated and the isotropic hyperfine couplings of the β-protons of the methyl group and the methylene group are found to be significantly smaller than those obtained by experiment (Table 1). For the optimized protonated radical II, the isotropic hyperfine couplings are quite close to those found experimentally (Table 1: 18.7 G (PCM) *vs*. 18 G (experimental) and 35.9 G (PCM) *vs*. 37 G (experimental)).

The poor fit of the theoretically predicted hyperfine coupling constant values of the anion radical (I) with the experimentally obtained ones and the excellent fit of the protonated radical (II) with experiment indicates that the carbonyl functional group protonates even at low temperatures in the glassy system. Calculations employing PCM do alter hyperfine couplings of the anion radical (I) marginally but have a negligible effect on hyperfine couplings of the protonated radical (II) (Table 1). The protonated carbonyl is predicted to show anisotropic proton hyperfine couplings but of a magnitude that would simply broaden the line components in the ESR spectra somewhat and this is in agreement with experiment (Section 2.1 and Figure 1).

**Figure 3:**
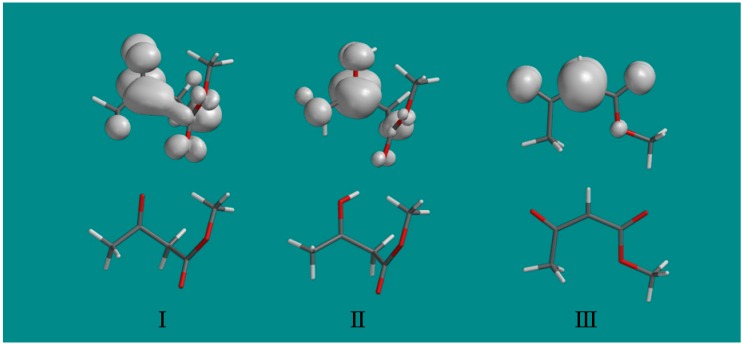
The spin density distributions of radicals I, II and III are shown above the optimized structure of each radical. Note that protonation of the oxygen at the carbonyl group of radical I substantially localizes the spin at the carbonyl group in radical II and increases the coupling to the methyl protons. Only a fixed methyl group is shown with spin density largely on one methyl proton, however, experimentally the methyl group rotates and averages the spin over the three protons.

For the final radical III, the only *ca.* 22 G doublet from the methylene α-proton is observed. The calculation provides a typical anisotropic proton coupling tensor with a *ca.* 21 G middle component that is in excellent agreement with the experimentally obtained overall value (Table 1, Figure 1). The spin density distributions in the three radicals found in this work are shown in Figure 3. They clearly show that on protonation of radical I to radical II, the unpaired spin localizes on the carbonyl group and substantially increases the interaction with the methyl group as indicated by the hyperfine couplings in Table 1. The final radical III shows the expected spin localization at the methylene carbon radical site.

## 3. Experimental Section

### 3.1. Materials

Methyl acetoacetate and deuterium oxide (D_2_O) (99.9 atom % D) were purchased from Sigma-Aldrich (St. Louis, MO, USA). Lithium chloride (LiCl, ultra dry, 99.995% (metals basis)) was obtained from Alfa Aesar (Ward Hill, MA, USA). Suprasil quartz tubes (4 mm OD, Catalog No. 734-PQ-8, Wilmad Glass Co., Inc., Buena, NJ, USA) were used for preparation of samples.

### 3.2. Sample Preparation

Approximately 6 milligrams (50 mM) of solute was dissolved in 7.5 M LiCl in H_2_O or D_2_O (1.0 mL). After the solute is fully dissolved, the solution has been bubbled with nitrogen gas for 5 min to remove oxygen from the samples. Subsequently, the solution is drawn into a 4 mm quartz tube and rapidly cooled to 77 K in liquid nitrogen to form a clear transparent glass as in our earlier works [8,9,10,11,13,14,15,16,18,19,20].

### 3.3. Gamma Irradiation

Gamma irradiations were performed with a GR-9 (Co-60) irradiator (dose rate = 0.5 kGy/h, absorbed dose = 600 Gy). All samples have been irradiated at 77 K in Teflon containers in the dark as well as are stored in these containers in the dark.

### 3.4. ESR Studies

Following our earlier studies, immediately after γ-irradiation of the glassy sample at 77 K, its ESR spectrum is recorded at 77 K. Also, immediately after each annealing step, the sample is cooled to 77 K by its immersion in liquid nitrogen (77 K) and its ESR spectrum is recorded at 77 K which maximizes signal height and allows for comparison of signal intensities. A Century Series X-band (9.3 GHz) ESR spectrometer (Varian, Palo Alto, CA, USA) with an E-4531 dual cavity, 9-inch magnet, and a 200 mW Klystron has been used. Fremy’s salt (*g*_center_ = 2.0056, *A*(N) = 13.09 G) has been employed for the field calibration. All ESR spectra have been recorded at 77 K and at 45 dB (6.3 μW).

### 3.5. Annealing of Glassy Samples and Radical Formation

Irradiation of the sample produces only prehydrated electrons and Cl_2_•^−^ [10,13,14]. Since only electrons are mobile at 77 K, all solute radicals at 77 K are formed due to the prehydrated electron attachment to the solute. A variable temperature assembly has been employed which passed liquid nitrogen cooled nitrogen gas past a thermister and over the sample as described in our earlier studies.Annealing of the sample at temperatures of 140 K–170 K allows for molecular migration as the glass softens and the stepwise observation of thermally induced radical reactions of the electron adducts. At these temperatures, Cl_2_•^−^ also becomes mobile but is not able to oxidize the solutes chosen for this study [10]. Thus, only solute anion radicals originating from prehydrated electron attachment and the subsequent reactions of these anion radicals are investigated in this work.

### 3.6. DFT Calculations

DFT calculations were performed using B3LYP and the 6-31G* basis set which is known to provide good estimates of hyperfine couplings [1,14,18,19,22]. All geometries were fully optimized. In our calculations, the solvent has been treated by use of the integral equation formalism polarized continuum model (IEF-PCM) as implemented in Gaussian 09 [23]. Calculations were performed using the Gaussian 09 program set [23]. The spin densities in the radicals have been obtained employing the Spartan’10 program set [24] at the B3LYP/6-31G*//B3LYP/6-31G* level theory in the gas phase.

## 4. Conclusions

This work leads to the following salient conclusions:

(i) In MAA, the added electron localizes at the carbonyl group:

The combination of ESR and DFT calculations presented in this work clearly shows that in a molecule with a carbonyl and ester functionality separated by a methylene bridge, the site of prehydrated electron attachment is preferentially at the carbonyl group. However, the spin density distribution of the initial anion radical shows the spin is actually shared between the two sites with increased localization at the carbonyl occurring on protonation of the carbonyl oxygen. Thus, the difference in electron affinity (EA) of the two sites is not likely to be high but the greater p*K*_a_ of the carbonyl site in the MAA anion radical favors the localization of the electron at that site.

(ii) Prehydrated electron-induced CH_3_-O bond cleavage is not observed in MAA:

Our previous work showed that prehydrated electron addition to methyl acetate at 77 K initiated dissociative electron attachment (DEA) that resulted in CH_3_• by C-O bond cleavage, reaction (5) [9]. Our recent work with peptide methyl esters such as N-acetylalanylalanine methyl ester [10], prehydrated electron addition also results in CH_3_• formation at 77 K by C-O bond cleavage (DEA) at 77 K. Electron attachment in the latter species can be at any of three sites which are each localized species. However, only the attachment to the ester site induces CH_3_• formation. In this work we find that in MAA, a molecule with a carbonyl and ester functionality separated by a methylene bridge, the prehydrated electron attachment is at both groups as they form a delocalized spin system that localizes only on protonation of the carbonyl oxygen (Figure 3). The added delocalization protects MAA from DEA and formation of CH_3_•.

(iii) The protonated anion radical of MAA is a H-atom abstraction agent: 

This protonated radical (radical II) is found to be a hydrogen abstracting agent as in similar molecules [8,9] and selects the weak methylene C-H bond for abstraction resulting in radical III. Radical III is expected to dimerize.
